# Metabolic response of dolphins to short-term fasting reveals physiological changes that differ from the traditional fasting model

**DOI:** 10.1242/jeb.238915

**Published:** 2021-05-04

**Authors:** Dorian S. Houser, Davina Derous, Alex Douglas, David Lusseau

**Affiliations:** 1 National Marine Mammal Foundation, San Diego, CA 92106, USA; 2 School of Biological Sciences, University of Aberdeen, Aberdeen AB24 2TZ, UK; 3 National Institute of Aquatic Resources, DTU Aqua, Technical University of Denmark, Kgs. Lyngby 2800, Denmark

**Keywords:** Metabolome, Foraging disruption, Lipolysis, *Tursiops truncatus*, Dynamic network marker

## Abstract

Bottlenose dolphins (*Tursiops truncatus*) typically feed on prey that are high in lipid and protein content and nearly devoid of carbohydrate, a dietary feature shared with other marine mammals. However, unlike fasted-adapted marine mammals that predictably incorporate fasting into their life history, dolphins feed intermittently throughout the day and are not believed to be fasting-adapted. To assess whether the physiological response to fasting in the dolphin shares features with or distinguishes them from those of fasting-adapted marine mammals, the plasma metabolomes of eight bottlenose dolphins were compared between post-absorptive and 24-h fasted states. Increases in most identified free fatty acids and lipid metabolites and reductions in most amino acids and their metabolites were consistent with the upregulation of lipolysis and lipid oxidation and the downregulation of protein catabolism and synthesis. Consistent with a previously hypothesized diabetic-like fasting state, fasting was associated with elevated glucose and patterns of certain metabolites (e.g. citrate, cis-aconitate, myristoleic acid) indicative of lipid synthesis and glucose cycling to protect endogenous glucose from oxidative disposal. Pathway analysis predicted an upregulation of cytokines, decreased cell growth and increased apoptosis including apoptosis of insulin-secreting β-cells. Metabolomic conditional mutual information networks were estimated for the post-absorptive and fasted states and ‘topological modules’ were estimated for each using the eigenvector approach to modularity network division. A dynamic network marker indicative of a physiological shift toward a negative energy state was subsequently identified that has the potential conservation application of assessing energy state balance in at-risk wild dolphins.

## Abstract (precis)

**Summary:** The non-fasting adapted dolphin demonstrates physiological responses to food deprivation that integrate characteristics of the traditional fasting model with specializations observed in other fasting-adapted marine mammals.

## INTRODUCTION

The physiological response to food deprivation in mammals is characterized by changing substrate utilization to maintain energy demands and is commonly divided into three phases (Castellini and Rea, 1992; McCue, 2010; Wang et al., 2006). The initial phase of fasting varies by species but starts immediately after the animal is post-absorptive from its last meal and lasts typically no longer than several days. This phase is characterized by a depletion of available glucose reserves (e.g. glycogen stores), a gradual reduction in the resting metabolic rate, and transition to lipid as the prime fuel substrate. The second phase is characterized by a reliance on lipid to meet most energy needs, a sustained increase in the mobilization of non-esterified fatty acids (NEFAs), negligible glucose oxidation, and a reduction in the catabolism of protein to spare lean tissue. The second phase of fasting is typically the longest endured and mass loss is slowed as energy-rich fat depots are depleted. The final phase of fasting occurs when usable fat stores are depleted and an increase in protein catabolism occurs to meet metabolic demands.

Phocids (earless seals) are a carnivorous taxon containing a number of species that have coupled fasting with breeding and lactation and engage fasts lasting from days to weeks or months. The phocid fasting response has been well characterized across a number of species but is probably best characterized in northern elephant seals (*Mirounga angustirostris*; Adams and Costa, 1993; Crocker et al., 1998; Houser and Costa, 2001; Houser et al., 2012; Keith, 1984; Ortiz et al., 1978; Pernia et al., 1980). The northern elephant seal engages in long-duration fasts during which it demonstrates a muted insulin response, adipose tissue resistance to insulin (but persistent sensitivity in muscle beds), high levels of circulating fatty acids, elevated levels of blood glucose, high rates of endogenous glucose production that rely little on amino acid precursors or glycerol (an end-product of fat catabolism), and high rates of pyruvate and tricarboxylic acid cycling (Champagne et al., 2005, 2006; Crocker et al., 2014a; Fowler et al., 2007; Houser et al., 2007, 2013; Kirby and Ortiz, 1994; Tavoni et al., 2013; Viscarra et al., 2013). Prolonged fasting in elephant seals is also associated with upregulation of the renin–angiotensin system, reduced expression of angiotensinogen in muscle and blubber, increased expression of TNF-α in muscle, reduced expression of blubber adiponectin, decreased and increased expression of hormone-sensitive lipase and adipose triglyceride lipase, respectively, and increased expression of AMP-activated protein kinases in adipose (Suzuki et al., 2013; Viscarra et al., 2011, 2012; Viscarra and Ortiz, 2013).

Unlike the phocids, little is known about fasting in cetaceans, and the few investigations that exist have been performed in a single species, the bottlenose dolphin (*Tursiops truncates*; Ortiz et al., 2010; Ridgway et al., 1970; Venn-Watson and Ridgway, 2007). In the wild, the bottlenose dolphin is generally considered an intermittent to continuous feeding animal that is not adapted to prolonged fasts. However, dolphins have been shown to maintain blood glucose at pre-fasting levels after 72 h of fasting, although variations in blood glucose, both increases and decreases, have been observed at shorter fasting intervals (Ortiz et al., 2010; Ridgway et al., 1970; Venn-Watson and Ridgway, 2007). Levels of NEFAs increase by 100% within 24 h of the onset of fasting concomitant with reductions in glucagon, a gluconeogenic hormone, and blood urea nitrogen (BUN) levels (Ortiz et al., 2010). Insulin levels were negligible in fasting dolphins, and dolphins submitted to oral, intraperitoneal or intravenous glucose tolerance tests showed prolonged glucose clearance curves ranging from 6 to 14 h, suggesting a muted insulin response or peripheral insulin resistance (Ortiz et al., 2010; Ridgway et al., 1970). Although fasted for shorter periods than is typical of many phocid fasts, the plasma metabolite patterns, hypoinsulinemia and muted insulin response to glucose challenges observed in the dolphin are qualitatively similar.

It has been hypothesized that phocid seals are pre-adapted to a fasting state because their prey are high in fat and protein and lack significant carbohydrate, i.e. the gross utilization of fuel substrates is similar between feeding and fasting states (Houser et al., 2007; Houser and Costa, 2001; Kirby and Ortiz, 1994). Dolphins and porpoises evolved under the same nutritional constraints and it is reasonable to hypothesize that odontocetes might also be pre-adapted to fasting. However, more detailed information on the odontocete fasting response is required, particularly given the potential for anthropogenic activity to disrupt odontocete foraging and the complex relationship foraging disruptions might have with changes in body condition (Derous et al., 2020). Understanding how odontocetes regulate energy homeostasis when food intake is disrupted is important to determining how human activities affect odontocete survival, growth and reproduction. In the marine environment, human activities can disrupt foraging either directly through competition (e.g. fisheries depletion) or indirectly by causing a behavioural response to the human activity (e.g. cessation of feeding behaviour or exclusion from feeding areas as might occur with some high-level acoustic activities). From a conservation perspective, it is therefore imperative that the impact of foraging disruption and how it influences individual contributions to population sustainability is understood (Derous et al., 2020; Pirotta et al., 2018).

Here, we investigated how the bottlenose dolphin, a non-fasting-adapted species, alters its metabolism in response to short-term fasting. Using an untargeted (‘shotgun’) metabolomics approach, we assessed a large number of plasma metabolites in a group of dolphins at two time periods using a paired design: when they were post-absorptive and following a 24-h fast. We provide an integrative insight of potential fasting-induced modifications to biochemical pathways, which can be compared with non-fasted- and fasted-adapted animals, as well as to other marine mammals that have also evolved on diets high in lipid and protein content but nearly devoid of carbohydrate. We conclude with insight on how techniques utilized in this study might be applied to conservation purposes in wild dolphins through identification of whether individuals are sampled (e.g. remote biopsy) while in a negative energy state.

## MATERIALS AND METHODS

### Dolphin subjects

Eight adult bottlenose dolphins [*Tursiops truncatus* (Montagu 1821)] were used in the study (7 males, 1 female; Table 1). The dolphins were maintained by the United States Navy Marine Mammal Program (MMP) at the Naval Information Warfare Center (NIWC) Pacific in San Diego, California. All procedures were approved by the Institutional Animal Care and Use Committee of NIWC Pacific, and each of the dolphins was declared fit for participation in the study by the MMP veterinary staff. The study followed all applicable US Department of Defense guidelines for the care and use of animals in research. Each of the dolphins was housed in netted enclosures within San Diego Bay. Dolphins were transported to above-ground pools for the experiment. On the day of transport, the dolphins were provided 1.8 kg of fish as a positive reward for voluntarily beaching and transporting to the pool. Three hours following the feeding, which ensured that the dolphins were post-absorptive (Yeates and Houser, 2008), each dolphin voluntarily presented its flukes for the collection of a blood sample. The sample was collected from the venous plexus of the ventral fluke with a 21 g butterfly needle. All samples were drawn into chilled EDTA plasma tubes (Becton Dickinson, Franklin Lakes, NJ, USA). Samples were centrifuged immediately after collection at 1090 ***g*** for 10 min, the plasma was collected, and samples were placed into a −80°C freezer. Time between sample collection and freezing was less than 20 min for all samples. The dolphins were then maintained under observation but were not provided any further food for a period of 24 h. At that time, a second blood collection was made and processed exactly as described for the post-absorptive sample. Following the collection, the dolphins were once again fed according to their normal feeding schedule.

**Table 1.**
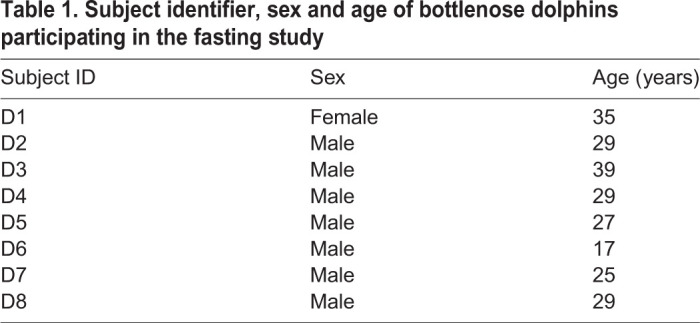
Subject identifier, sex and age of bottlenose dolphins participating in the fasting study

### Untargeted metabolomics

Plasma samples were shipped on dry ice to Metabolon (Metabolon Inc., Durham, NC, USA) to be processed for a suite of metabolites on gas chromatography–mass spectrometry (GC/MS) and liquid chromatography and tandem mass spectrometry (LC/MS/MS) platforms. The processing procedures used by Metabolon have been published previously and the reader is referred to these articles for a detailed methodology (Evans et al., 2009; Sha et al., 2010). In brief, recovery standards were added to each sample for quality control purposes. Samples were then extracted in a series of proprietary organic and aqueous solvents and split into two fractions. The solvents in each fraction were removed by roto-evaporation and the samples were frozen under vacuum. A small amount of each sample was pooled to use as a technical replicate that was run periodically throughout processing to assess process variability.

A Waters Acquity ultra-performance liquid chromatograph (Waters Corp., Milford, MA, USA) and a Thermo-Finnigan LTQ MS (Thermo Finnigan LLC, San Jose, CA, USA) with an electrospray ionization source and a linear ion-trap mass analyzer were used for LC/MS analysis. Sample extracts were divided into aliquots and reconstituted in basic (6.5 mmol l^−1^ ammonium bicarbonate, pH 8) or acidic (0.1% formic acid) solvents. Into each of these, 11 standards were added at known quantities. Acidic preparations were eluted using a gradient elution from 99.5% water/0.5% methanol to 1% water/99% methanol using water and methanol containing 0.1% formic acid, while in a separate analytical run basic preparations were eluted using a gradient elution from 99.5% water/0.5% methanol to 1% water/99% methanol with a consistent 6.5 mmol l^−1^ concentration of ammonium bicarbonate. Both methods were operated with a 350 µl min^−1^ flow rate.

The column used for GC analysis was 5% phenyldimethyl silicone. The temperature profile for the GC analysis started at 40°C and increased to 300°C over 16 min, and the carrier gas for the column was helium. Mass spectrometry was performed with a Thermo-Finnigan Trace DSQ fast-scanning single quadrupole MS using electron impact ionization. Sample aliquots for GC/MS were derivatized with equal parts of bistrim-ethyl-silyl-trifluoroacetamide and a mixture of acetonitrile, dichloromethane and cyclohexane at a volume ratio of 5:4:1. Derivatization was performed in the presence of 5% triethylamine for 1 h at 60°C.

Peak integration was performed with Metabolon's proprietary software and compounds were identified by comparison of chromatographic properties and mass spectra against 1500 commercially available and purified biochemicals. Peak integration and compound identification were managed in Metabolon's Laboratory Information Management System.

### Metabolomic profile analysis

Peak integration was utilized as a measure of ‘metabolite expression’. A hierarchical model was fit to metabolite expression using empirical Bayes moderation to estimate individual variance and linear models to estimate change in metabolite expression given the paired design of the study using the package metabolomics (based on limma)in R v.3.5 (https://rdrr.io/cran/metabolomics/). The *P*-values were adjusted using Benjamini–Hochberg's approach to account for multiple testing. Following this univariate approach to differential metabolite expression, a discriminant function was fitted to all metabolites to determine whether the treatment levels could be discriminated based on metabolite profiles, and if so, to determine which metabolites had a predominant role in this profiling. This was done by defining a function based on two principal components of the variance in metabolite expression, taking the paired design into consideration using a partial-least square approach to discriminant analysis (PLS-DA) using the mixOmics package in R (Rohart et al., 2017).

### Metabolomic network topological changes

The main axes of the study results were estimated by decomposing the 3D data tensor (322 metabolites×8 individuals×2 treatment levels) using higher-order singular value decomposition (HOSVD; Kolda and Bader, 2009). HOSVD provides a way to understand the main matrices of variance in the data; in this case, deriving principal components of the between-treatment level variance among metabolites as well as the overall contribution of metabolites to the tensor covariance.

The physics of molecular networks – a complex web of relationships between molecules – provides important insights about the dynamical biological changes that take place during a physiological perturbation. The architecture – or topology – of the networks can reveal: (i) how biological pathways interact and how these interactions change under perturbation (Jin et al., 2020); (ii) which metabolite(s) play a predominant role in the network, regardless of their differential expression (Derous et al., 2016); and (iii) the precursor of state shifts [dynamical network marker (DNM)], which can help diagnose phenotypically important shifts before they occur (Liu et al., 2019; Zeng et al., 2013). Here, we investigated how the metabolomic network changed in response to fasting. Metabolomic conditional mutual information (CMI) networks were estimated for each treatment level using the between-individual metabolite expression co-variance (estimated using mutual information) as a proxy for interaction between metabolites. To remove indirect effects caused by many metabolite pairs interacting, mutual information of pairs were conditioned on the mutual information they shared with other metabolites. As the networks were small, this was implemented using the aracne method in the minet package in R (Margolin et al., 2006).

How metabolites clustered in the networks as ‘topological modules’ was estimated using the eigenvector approach to the modularity network division. In short, the modularity approach (and its associated coefficient, *Q*) to divide networks into clusters is motivated by the notion that clusters should group more closely with metabolites that have higher co-expression and less closely with those that are not part of their group (Spirin and Mirny, 2003). To determine whether network topology changed in relation to fasting, changes in metabolite module membership after fasting were assessed. Specific metabolites that predominantly contributed to the overall topology of the network were identified by estimating their eigenvector centrality (Newman, 2018). Finally, we estimated whether a DNM emerged during this short fasting period. Prolonged fasting, or calorie restriction, induces important phenotypic state shifts and these shifts can be reflected by a change in gene-gene network structure (Derous et al., 2016). Consequently, a DNM was expected to emerge as a precursor to the prolonged state shift. To detect this, changes in between-module variability in eigenvector centrality prior to and after fasting were estimated with a general linear model (GLM), as it was expected that the network would re-arrange itself around the DNM during its formation (Liu et al., 2019). As the network approaches state-shift, an increased stochasticity in the expression of metabolites involved in the DNM is also expected (Liu et al., 2015). Following the central limit theorem, between-individual variance in metabolite expression was used as an estimate of the stochasticity of metabolite expression (a higher between-individual variance is an estimate of a higher metabolite expression variance) and to determine whether this variance (coefficient of variation) was associated with DNM module membership and larger for the candidate DNM module.

### Inferring changes in biological pathways and functions

The information that could be inferred from the differential expression in metabolites was overlapped with known relationships and biological functions of those metabolites. Inferences were made about likely changes in biological pathways and functions given the observed changes in metabolite expression. This was first done by grouping and relating metabolites to pathways identified in the KEGG pathways database. Then, relationships to canonical pathways through Ingenuity Pathway Analysis (IPA; QIAGEN Inc., https://www.qiagenbioinformatics.com/products/ingenuity-pathway-analysis) was used to estimate inhibition and activation of canonical pathways and biological functions via the Molecule Activity Predictor (MAP) function (based on an IPA proprietary curated knowledge database). This allowed visualization of metabolic changes by simulating directional consequences on downstream molecules and inferred activity upstream from the metabolite. This enabled predictions about upstream regulator activity given the metabolomic information. However, it is important to highlight that IPA has limitations in that molecular studies of human health are overrepresented in its proprietary library. This creates potential for the overrepresentation of particular processes involved in cell growth and apoptosis (oncology). It is also likely that large subpathways less conserved across species will be under-represented in IPA regulatory activity analyses. Predictions for downstream and upstream changes in signalling molecules given the metabolomic profiles were also derived. To do so, the log-fold change estimate and its associated adjusted *P*-value for known metabolites was related to its biological function involvement as experimentally demonstrated (i.e. using the human metabolome database for identification, the KEGG pathway database, and proprietary IPA databases for known relationships and pathway inference). This allowed assessment of metabolite interactions and the potential impact each metabolite might have on a pathway level.

## RESULTS

### Metabolomic profiling

**Fig. 1.**
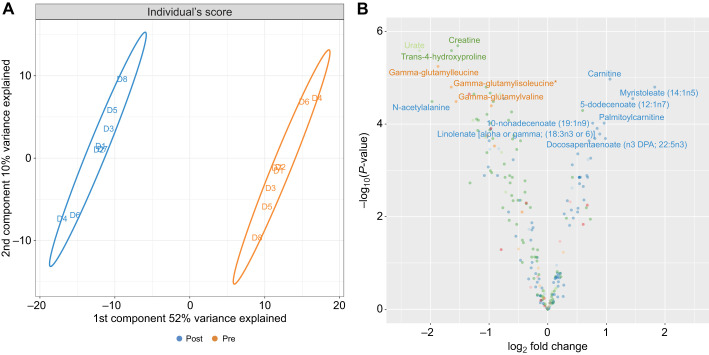
**Discrimination of experimental groups and component metabolites with the largest fold changes between groups.** (A) Partial least square-discriminant analysis (PLS-DA) score plot for each post-absorptive and fasted individual. Ellipses correspond to 95% of the region occupied by each group defined by the discriminant function. Groupings correspond to the treatment levels (orange for post-absorptive and blue for fasted) and are substantially differentiated in the metabolite space by the first two principal components of the 322-metabolite intensity matrix. (B) Volcano plot of metabolite log_2_ fold change (*x*-axis) and associated adjusted *P*-value (*y*-axis), accounting for the paired design and multiple testing [–log_10_(*P*-value)]. The known metabolites with the largest log-fold changes (LFC) are identified (LFC smaller than the 2.5% or larger than the 97.5% quantiles of all LFC). Colors correspond to the main metabolic ‘super pathways’ to which metabolites belong as described in the legend of Fig. 2.

A total of 322 metabolites were isolated in the dolphin plasma samples. Of these, 219 were positively identified by reference to known biochemicals within the available metabolite libraries. The remaining 103 compounds were of undetermined structural identity. After accounting for the paired study design using the hierarchical model and adjusting for multiple tests with Benjamini–Hochberg's correction, 150 of the metabolites had significantly different expression after fasting (Table 2, Fig. 1B, adjusted *P*-values <0.05). The ratio of group means for select identified metabolites and their respective intensity ratios (intensity_fasted_/intensity_post-absorptive_) are shown in Tables S1 and S2.

**Table 2.**
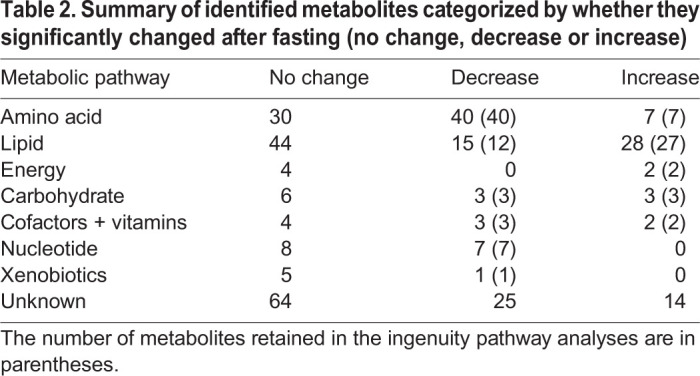
Summary of identified metabolites categorized by whether they significantly changed after fasting (no change, decrease or increase)

**Fig. 2.**
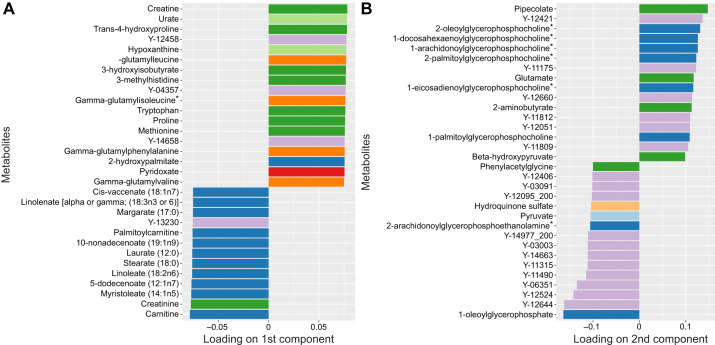
**A subset of largest loadings (loadings >95% and <5% of the quantiles of the loadings retained) of metabolites on principal components of the PLS-DA.** (A) PC1; (B) PC2. For both panels, metabolites designated with ‘Y-xxxx’ correspond to detected but unidentified compounds of the metabolome (i.e. they could not be assigned to a super pathway). Colors correspond to the main metabolic ‘super pathways’ to which metabolites belong, as follows: dark green, amino acid; light green, nucleotide; dark blue, lipid; light blue, carbohydrate; dark red, cofactors and vitamins; light red, energy; dark orange, peptide; light orange, xenobiotics; purple, unidentified.

The log_2_ fold change in intensity varied from −2.5 to 2.5, and there was a marked difference in the metabolic profile of the dolphin plasma after fasting (Fig. 1B). The two principal components of the discriminant function, which explained respectively 52% and 10% of the variance in metabolite intensity (Fig. 1A), were primarily associated with metabolites involved in amino acid and lipid metabolism (Fig. 2). Metabolites associated with lipid metabolism increased in expression with the fasted profile while metabolites associated with amino acid metabolism decreased. Creatine and urate had the largest loadings in concordance with the post-absorptive state while creatinine and carnitine had the largest loadings relating to the fasted state.

The HOSVD of the data tensor highlighted that urea, phosphate, lactate and glucose contributed most to the variance of the tensor (Fig. S1A), meaning that changes in their co-variance contributed substantially to the overall change in the plasma metabolomic signature. The change was not associated with between-individual variance in metabolites (Fig. S1B) but more with the treatment effect (Fig. S1D), even though intensity values did not change substantially between treatment levels (Fig. S1C).

### Metabolomic network topological changes

The topology of the metabolomic network, a network of metabolite CMI for each treatment level, changed after fasting (Fig. S2). While the modularity of the network did not change substantially (*Q*_post-absorptive_=0.47, *Q*_fasted_=0.46), the way in which metabolites clustered did (Fig. S3). The metabolites with the greatest eigenvector centrality changed between the two networks from the γ-glutamyl amino acids in the post-absorptive state to the glycerophosphocholines in the fasted state, although none of the structurally important glycerophosphocholines identified were differentially expressed.

**Fig. 3.**
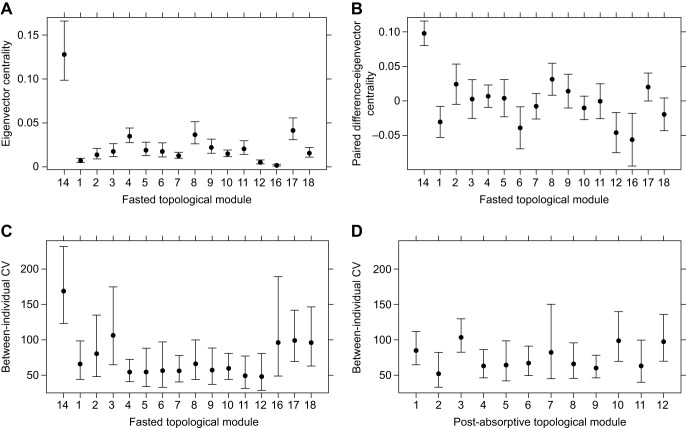
**Eigenvector centrality, paired difference-eigenvector centrality, and between-individual CV for group-dependent topological modules.** (A) Predicted eigenvector centrality of metabolites for each fasted topological module [back-transformed from a GLM fitted to log(eigenvector centrality estimate)] and (B) paired change in eigenvector centrality depending on metabolite membership of fasted topological modules. Predicted (C) fasted and (D) post-absorptive between-individual CV in metabolite intensity for each module [back-transformed from a GLM fitted to log(CV)]. Error bars are 95% confidence intervals. Fasted topological modules 13 and 15 only had one metabolite and so were removed for the analyses.

A module emerged in the fasting state with the largest modularity coefficient (Fig. S3) and regrouped all metabolites with the largest eigenvector centrality (module 14; Fig. 3). Eigenvector centrality varied significantly between modules (GLM, *F*_15,304_=28.5, *P*<0.0001). The metabolites in this candidate DNM module (Tables S3) had higher eigenvector centrality (Fig. 3A), and it was the module for which metabolite eigenvector centrality changed the most (Fig. 3B) with paired differences varying significantly between modules (GLM, *F*_15,304_=10.8, *P*<0.0001).

The candidate DNM module (module 14, Fig. 3) had the greatest between-individual coefficient of variation in metabolite intensity (*F*_15,304_=252.1, *P*<0.0001; Fig. 3C). Whilst the mean within-module CV was greatest for the DNM, it was not different from module 3 and 16 (mainly because those modules had large variability in CV, Fig. 3C). Such marked heterogeneity in CVs between modules was not observed in the post-absorptive state (*F*_11,310_=1.9, *P*=0.03; Fig. 3D; driven by the pairwise difference between modules 2 and 3). These features characterise the dynamic process the network underwent, including the re-assortment of its topology around the DNM.

### Inferring changes in biological pathways and functions

**Fig. 4.**
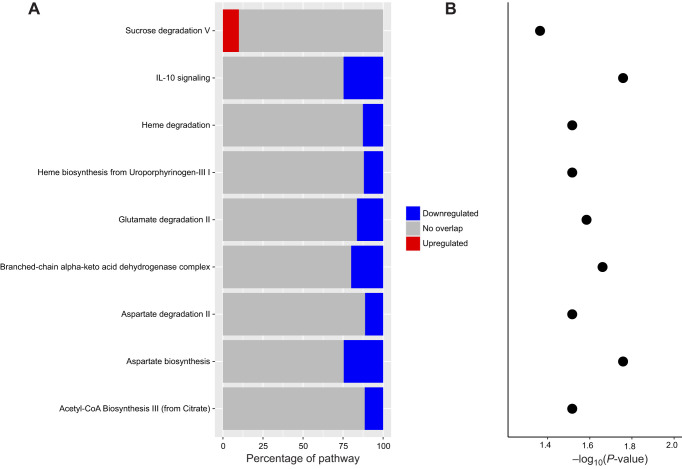
**Canonical pathways predicted (from the DNM) to be affected given the change in metabolite expression observed while fasting.** (A) The percentage corresponds to the percentage of genes in the pathway predicted to be upregulated or downregulated given the metabolite profile of the samples. (B) The –log_10_(*P*-value) of the Fisher exact test determining the likelihood that the association between the set of identified metabolites and the pathway is due to chance. Significant values (>1.3) identify non-random associations.

**Fig. 5.**
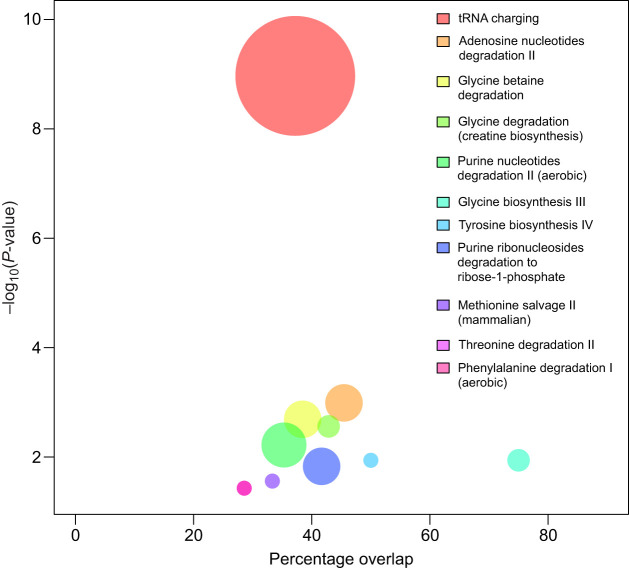
Bubble plot of the overlap of metabolites changing intensity after fasting and biological pathways in relation to the significance of the differential change in intensity of the pathways [–log_10_(*P*-value)].

**Fig. 6.**
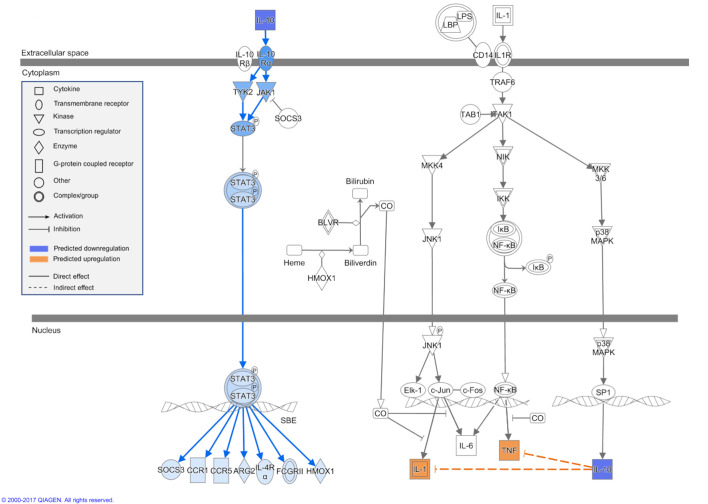
**Schematic of the predicted effect of IL-10 signalling** **downregulation.** The networks and figures were generated through the use of IPA (QIAGEN Inc., https://www.qiagenbioinformatics.com/products/ingenuity-pathway-analysis).

Metabolites that significantly changed in expression, i.e. discounting the glycerophosphocholines, overlapped significantly with several canonical pathways (Table S1). A decrease in activity with fasting was inferred for most canonical pathways, particularly IL-10 signalling and pathways linked to amino acid biosynthesis (via aspartate and glutamate; Fig. 4). With regard to amino acid biosynthesis, the most striking difference between the post-absorptive and fasted state was an inferred decrease in tRNA charging (Fig. 5). The DNM identification suggested IL-10 signalling downregulation after fasting (Fig. 6), which could not be inferred from single metabolite analyses alone.

Using the log-fold change estimate approach and arbitrarily setting a *Z*-score of <–2 or >2 to indicate pathways inferred to have changed the most, the pathways predicted to be most affected (Table S2) were predominated by a decrease in efflux and an increase in uptake of amino acids, a decrease in cell growth and survival, and an increase in apoptosis. To a lesser degree (–2<*Z*<–1 or 1>*Z*>2), changes in lipid metabolism functions suggested an increase in the storage of triacylglycerol (recalling that blood was sampled) and increased availability of lipids.

**Fig. 7.**
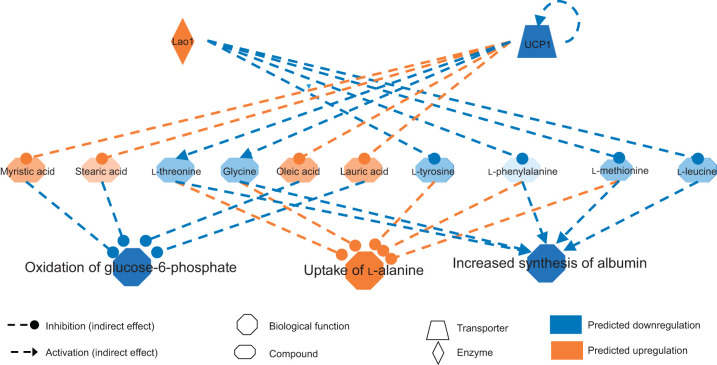
**Regulatory network predicted from pathway changes following fasting as estimated by ingenuity pathway analysis.** Blue lines and nodes correspond to pathway inhibition whereas orange lines and nodes correspond to pathway activation.

By using the molecule activity predictor (MAP) function, predictions about changes likely to occur upstream and downstream from the direct observations were made. The function predicted that the observed changes in metabolite expression observed would be associated with an upregulation of cytokines, decreased cell growth, and increased apoptosis including apoptosis of insulin-secreting β-cells. The upstream analyses predicted three regulators to be activated (Lao1, sirolimus, afatinib) by fasting and one inhibited (UCP1), all putatively involved in cell growth and apoptosis (Fig. 7). This suggests decreased glucose-6-phosphate oxidation and therefore decreased nucleotide anabolism via the pentose phosphate pathway. It also leads to increased uptake in l-alanine, likely linked to a decrease in gluconeogenic activities, concordant with changes in urea (Fig. S1), and predicts a decreased synthesis of albumin that may reflect the binding of albumin to fatty acids for oxidation.

## DISCUSSION

The majority of the variance in metabolite intensities between the post-absorptive and 24-h fasted states, as described by the two dominant principal components, were related to amino acid and lipid metabolism. Specifically, dolphins demonstrated a metabolic shift characterized by significant increases in circulating free fatty acids and significant reductions in nearly all metabolites associated with amino acid metabolism. Patterns in circulating urea and free fatty acids mirrored that reported for dolphins previously fasted for up to 38 h (Ortiz et al., 2010). The pattern is consistent with metabolic shifts toward lipolysis and lipid oxidation to meet energy needs and away from protein oxidation, and is consistent with observations in other fasting mammals and with prior observations in short-term fasted dolphins (Ortiz et al., 2010). However, the pattern differs from the typical mammalian response in several ways. These are potentially shared by other marine mammals and might reflect a commonality in fasting responses that diverges from the traditional terrestrial model.

### Changing energy state

Creatine and creatinine shared the largest loadings on the principal components for the post-absorptive and fasted states, respectively. The shift in these interconverting compounds, with creatine decreasing during fasting and creatinine increasing, potentially relate to changing intracellular energy demands, particularly in slow-twitch muscle or heart (Wyss and Kaddurah-Daouk, 2000). Creatine production might decline without exogenous amino acid availability, yet an increase in cellular uptake could also explain the decline in plasma creatine, as has been observed in some animal models of starvation (e.g. Zhao et al., 2002). Creatinine is formed intracellularly, either from phosphocreatine (PCr) or creatine, and diffuses into the blood once formed. Reduction in creatine kinase activity, which regulates the phosphorylation of creatine, would facilitate non-reversible degradation to creatinine while diminishing energy transport capacity, i.e. the transport of high-energy phosphates (Wyss and Kaddurah-Daouk, 2000). The proposed mechanism must consider that an increase in creatinine is often associated with muscle wasting or reflective of a reduction in the glomerular filtration rate (GFR; Feher, 2017). However, urea also increases when protein oxidation increases and would be expected to be at higher levels in the plasma, which was not observed after 24 h of fasting. An indication of reduced GFR would also require a disproportionately greater increase in urea than in creatinine, the so-called U:C ratio, but this was not observed. Rather, the patterns in urea and creatinine are consistent with protein conservation patterns observed in fasting-adapted marine mammals and bears (Adams and Costa, 1993; Chow et al., 2011; Derocher et al., 1990; Houser et al., 2001; Nelson et al., 1984; Ramsay et al., 1991). Thus, a reduction in urea with fasting, limited evidence for hemoconcentration of other metabolites, and a profile indicative of protein sparing (see below) suggests that these are not primary drivers of increased plasma creatinine and that plasma elevations in creatinine and reductions in plasma creatine may be indicative of a downregulated energy state.

### Amino acid metabolism

The vast majority of amino acids and metabolites declined with fasting, as did the end-product of amino acid catabolism, urea, its cycle intermediates, and purine catabolism end-products (urate and allantoin). Much fewer showed no significant change and only five of the 77 metabolites identified and associated with amino acid metabolism increased with time fasting. One of them, creatinine, has been discussed. The overall pattern is consistent with the protection of protein stores from oxidation. Because charged tRNA carries activated amino acids to the ribosome to synthesize protein (Raina and Ibba, 2014), the inferred decrease in tRNA charging (Fig. 5) suggests downregulation of protein synthesis. This is supported by the decline of all γ-glutamyl amino acids with fasting (Table S1). Collectively, the reductions in protein oxidation and synthesis patterns are consistent with the traditional fasting model of conserving endogenous protein stores.

Histidine levels did not significantly change with fasting, but significant reductions in 1-methylhstidine and 3-methylhistdine and a significant increase in 1-methylimidazoleacetate suggest that fasting, and maybe more specifically the lack of dietary protein, induced a change in histidine metabolism that favoured upregulation of the histamine pathway. Evidence suggests mast cells increase histamine release into the portal circulation during fasting, which promotes ketogenesis by upregulating production of oleoylethanolamide in the liver (Misto et al., 2019). The significant increase in 1-methylimidazoleacetate, the catabolic end-product of histamine degradation, suggests an increase in histamine flux toward degradation. However, without measuring flux rates or at least knowing changes in histamine levels relative to metabolites in the same pathway, it cannot be discerned whether histamine levels are elevated or whether a more rapid degradation maintains or reduces levels during fasting. No evidence of ketosis was identified in this study, but prior work suggests that ketone levels decrease over a period of up to 72 h of fasting in dolphins (Ridgway, 1972). Minimal ketone production and lack of ketoacidosis is also a hallmark of the fasting-adapted seals (Castellini and Costa, 1990; Crocker et al., 2014b; Nordøy and Blix, 1991), suggesting some potential commonality between the fasting response of dolphins and seals. How the suppression of ketosis with fasting might relate to histidine/histamine metabolism, however, requires further investigation.

### Lipid metabolism

Dolphins fasted for 24 h demonstrated a significant increase in circulating FFA including long-, medium- and short-chained fatty acids of both saturated and unsaturated forms. All but three FFA identified showed a fold change >1.0 (range 1.13–6.43). Along with a significant increase in glycerol, the pattern is consistent with a fasting-induced upregulation of lipolysis, which has previously been observed in fasting dolphins (Ortiz et al., 2010). The increase in lipolysis presumably increases the intramyocellular content of muscles in order to reduce glucose oxidation in the absence of feeding, as has been observed in fasted, obese humans (Høgild et al., 2019). Consistent with this, the significant increase in carnitine suggests an increase in the β-oxidation of fatty acids that defends other fuel substrates from oxidation. Prior measurements of the respiratory quotient (RQ) in a fasting dolphin yielded an RQ=0.73, which is close to optimal for lipid oxidation and is consistent with evidence presented here (Pierce, 1970).

All of the FFA that demonstrated significant increases with fasting, excepting caprate (10:0) and isovalerate (5:0), were long-chain fatty acids (LCFAs, number of carbons >12) and equated to 91% of the significantly increased fraction of FFAs. Most of the identified FFAs in circulation have previously been identified in the blubber of dolphins and other small odontocetes and have demonstrated differential concentration depending upon the depth of the blubber strata (Koopman et al., 1996; Samuel and Worthy, 2004). Myristoleate (14:1n5) and 5-dodecenoate (12:1n7) by far showed the greatest increases in plasma with fold changes of 6.43 and 4.46, respectively (the fold changes of all other FFAs were <3.00). Myristoleate is not found in significant quantities in most fish, but it has been identified in cetacean and pinniped blubber suggesting that endogenous synthesis might have converged across the marine mammals; in dolphins, accumulation appears to occur in the less metabolically active outer blubber layer (Ackman and Lamothe, 1989; Grahl-Nielsen et al., 2010; Iverson et al., 1995; Koopman et al., 1996; Samuel and Worthy, 2004; Smith and Worthy, 2006). The fasting bottlenose dolphin likely exhibits an active Δ9-desaturase system for myristoleate synthesis (e.g. Mosley and McGuire, 2007), possibly within either the deep blubber layer or in the liver, but with subsequent mobilization into the plasma pool. Myristoleate synthesis may be a conserved response to fasting across mammals as it has been observed to increase in the adipose tissue of humans under dietary restriction (Montastier et al., 2015) and in the plasma of circannual hibernators at the point of arousal (Epperson et al., 2011). However, the metabolic fate of both myristoleate and dodecanoate is ultimately unresolved.

### Carbohydrate metabolism

A decrease in circulating glucose levels is observed in humans and laboratory mammals within 24 h of fasting, yet a slight increase in circulating glucose levels was observed in the dolphins fasted here. Increases in circulating glucose have previously been observed in dolphins fasted for durations of up to 72 h (Ortiz et al., 2010; Ridgway, 1972; Ridgway et al., 1970; Venn-Watson and Ridgway, 2007), although acute decreases in glucose levels were noted in some cases prior to later stabilization at post-prandial or higher levels. Elevated levels of fasting glucose have been hypothesized to be related to a diabetes-like physiological state (Venn-Watson and Ridgway, 2007), and a similar hypothesis has been proposed for fasting pinnipeds that also demonstrate elevated fasting serum glucose (see Houser et al., 2013). Glucose tolerance tests in dolphins show prolonged glucose clearance rates suggestive of peripheral insulin resistance, similar to that observed in elephant seals (Fowler et al., 2008; Viscarra et al., 2011). However, dolphins show an increase in serum glucose for several hours after feeding with a concomitant rise in insulin that is dependent on the amount of protein ingested (Venn-Watson et al., 2011). The results suggest the activation of amino acid-based gluconeogenic pathways when dietary protein is available and possible amino acid signalling of insulin production. As such, insulin levels would presumably be maintained at low levels while fasting, and if combined with any degree of peripheral insulin resistance, would contribute to sustained and elevated glucose levels.

Endogenous glucose production (EGP) in a fasting dolphin has not been reported, although glucose oxidation appears low as determined from calculation of the fasting RQ (Pierce, 1970). Fasting northern elephant seals also show a fasting hyperglycemia and low rates of glucose oxidation (Houser et al., 2012). These have been related to high rates of EGP and underlying high rates of tricarboxylic acid and pyruvate cycles (Champagne et al., 2005, 2006, 2012). In these fasting-adapted seals, protein, glycerol and glycogen make negligible contributions to EGP, although the glycerol contribution appears sufficient to account for the small amount of glucose that is oxidized (Champagne et al., 2012; Houser et al., 2007; Houser and Costa, 2001). Gluconeogenesis appears to be part of a futile cycle with phosphoenolypyruvate (PEP) as the primary gluconeogenic precursor that draws from the available lactate pool (Champagne et al., 2012; Tavoni et al., 2013). Changes in glucose and lactate between post-absorptive and fasted states suggest a similar system might exist in the fasted bottlenose dolphin. The system would require a greater flux of metabolites through the tricarboxylic acid cycle (TCA) cycle, which has been suggested as a means not to meet glucose demands, but to deal with the rapid upregulation of fatty acid β-oxidation while mitigating significant ketosis (Houser et al., 2012).

Invoking synthesis of triacylglycerols from glucose could further mitigate significant ketosis, which can be accomplished in both liver and adipose tissue by shunting glucose through the pentose phosphate pathway (PPP) and producing NADH+H^+^ for biosynthetic reactions (Salway, 1999). The condition for activation of this path is a high-energy state that inhibits isocitrate dehydrogenase, which subsequently produces an accumulation of citrate and isocitrate, the former diffusing into the cytosol via a tricarboxylate carrier. Citrate in the cytosol serves both as a substrate for fatty acid synthesis (being cleaved to form oxaloacetate and acetyl-CoA) and the activator of acetyl-CoA carboxylase, which is required for the synthetic reaction. Although isocitrate was not identified in the fasted dolphin serum metabolome, both citrate and cis-aconitate, a transitional compound in the isomerization of citrate to isocitrate, increased significantly in the fasted state whereas other TCA cycle intermediates did not. If observations of these compounds in the plasma are indicative of reduced isocitrate dehydrogenase activity and reflective of fatty acid synthesis, it would help keep glucose largely in non-oxidative pathways while simultaneously mitigating significant ketosis.

The issue with the aforementioned hypothesis is that utilization of the PPP would presumably increase glucose-6-phosphatase oxidation, which is contrary to the MAPs presented here. However, it must also be acknowledged that the IPA data upon which this prediction is based are biased toward molecular studies of human health, particularly oncological processes involved in cell growth and apoptosis; therefore, inferences that rely on MAPs of metabolic pathways in fasting dolphins might be misleading. A much less understood yet alternative pathway is mitochondrial fatty acid synthesis (mtFAS), in which glucose-derived acetyl-CoA is directed toward fatty acid synthesis once entering the mitochondria (Hiltunen et al., 2010). Occurring in the liver, mtFAS was historically deemed a means by which lipoic acid is generated, but it is now known that mtFAS can synthesize fatty acids with carbon chain lengths of up 14-16 carbon atoms (Hiltunen et al., 2010, 2009; Nowinski et al., 2018). In this context, it is interesting that the greatest fold change of all metabolites was in myristoleate (14:1n5). Although the fate of long-chain fatty acids produced via mtFAS is unknown and its regulatory mechanisms are poorly understood, the presence of such a mechanism could provide yet another means by which glucose stores are protected from oxidation.

### The dynamic network marker

Fasting bottlenose dolphins continue to show physiological changes associated with fasting up to 72 h after their last feeding (Ortiz et al., 2010; Ridgway, 1972). The DNM can be used to characterize a transition between physiological states, as should have been the case for the dolphins fasted for 24 h in this experiment. Short-term fasting triggered a re-arrangement of the dolphin blood metabolomic network, yielding a putative DNM that could be used as an early-warning signal of phenomic state-shift (Liu et al., 2015); in other words, a negative-energy transitional state pointing toward a new stable negative energy physiological state (i.e. stage 2 of fasting). Two conclusions can be reached from the detection of a DNM. First, a non-linear change in the blood metabolome of dolphins in response to a lack of food is expected with a state shift towards a predominant lipid-utilization phenome. Second, subsequent physiological changes associated with decreased growth and increased apoptosis would be expected.

The DNM highlights two important features of the dynamic metabolome that could not have been detected from univariate analyses alone. First, glycerophosphocholines (GPCs) were identified as important for the structure of the metabolomic network despite not being differentially expressed. This advantage of a network approach to transcriptomic and metabolomic expression has been highlighted before (Derous et al., 2016; Laye et al., 2015) and helps show that differential expression alone may not provide a full picture of metabolic dynamics. GPCs are early markers of insulin resistance and impaired glucose tolerance in the development of dysglycemia and type 2 diabetes in humans (Cobb et al., 2013; Ferrannini et al., 2013), and are associated with insulin resistance and β-cell dysfunction. This is consistent with the emergence from differential expression of pathways in the present study [e.g. significant reduction in nervonate, which has been associated with insulin resistance (Yamazaki et al., 2014)], previous experimental work (Venn-Watson et al., 2011) and comparative genomic studies (Derous et al., 2021) that suggest fasting bottlenose dolphins protect circulating glucose from oxidative pathways. IL-10 signalling was also well represented in the DNM and may be an early signaller to the JAK/STAT pathway. IL-10 primarily acts on the JAK/STAT pathway and the NF-κB pathway by increasing TNF and IL-1, in both cases leading to effects on cell growth, survival and apoptosis. As such, it indicates potential changes in the cell life cycle as a consequence of the dolphin's negative energy state.

The DNM could be a useful early metabolic marker of dolphins transitioning to a negative energy state, having to protect circulating glucose, and entering a state of decreased cell growth and survival and increased apoptosis. The fact that such marked differences can be detected in the metabolomic profile of genetically diverse, fasted dolphins hints at the potential for using such a DNM from samples collected from wild populations of bottlenose dolphins to evaluate whether they are shifting toward a negative energy state. The ability to detect the DNM from tissues that can be obtained remotely (e.g. blubber biopsies) could be useful for monitoring individuals in a population to assess whether natural or anthropogenic events affect energy acquisition (i.e. prey capture). The next step in making the marker efficacious would then be to determine how the transitional state, once identified, translates into an impact on reproductive effort/success or survival. Furthermore, how recurrent entrainment of the fasting response to repeated foraging disruptions, which has been highlighted as a key pathway by which human activities at sea can affect the conservation status of cetaceans, also needs to be determined.

## Supplementary Material

Supplementary information
