# Supplementary material for: Metabolic response of dolphins to short-term fasting reveals physiological changes that differ from the traditional fasting model
Source: J Exp Biol. 2021 May 4;224(9):jeb238915. doi: 10.1242/jeb.238915 (PMC8126448; doi:10.1242/jeb.238915)
Supplement: Supplementary information [file jexbio-224-238915-s1.pdf]

Figure S1. Eigensets of the HOSVD for those metabolites with (A) the largest contribution on the first eigenarray of the metabolite eigenmatrix: (B) metabolite x dolphin ID eigenset, (C) metabolite x treatment eigenset, and (D) dolphin ID x treatment eigenset. The metabolites Y-12051 and Y-11204 correspond to the Metabolon ID for unidentified compounds.

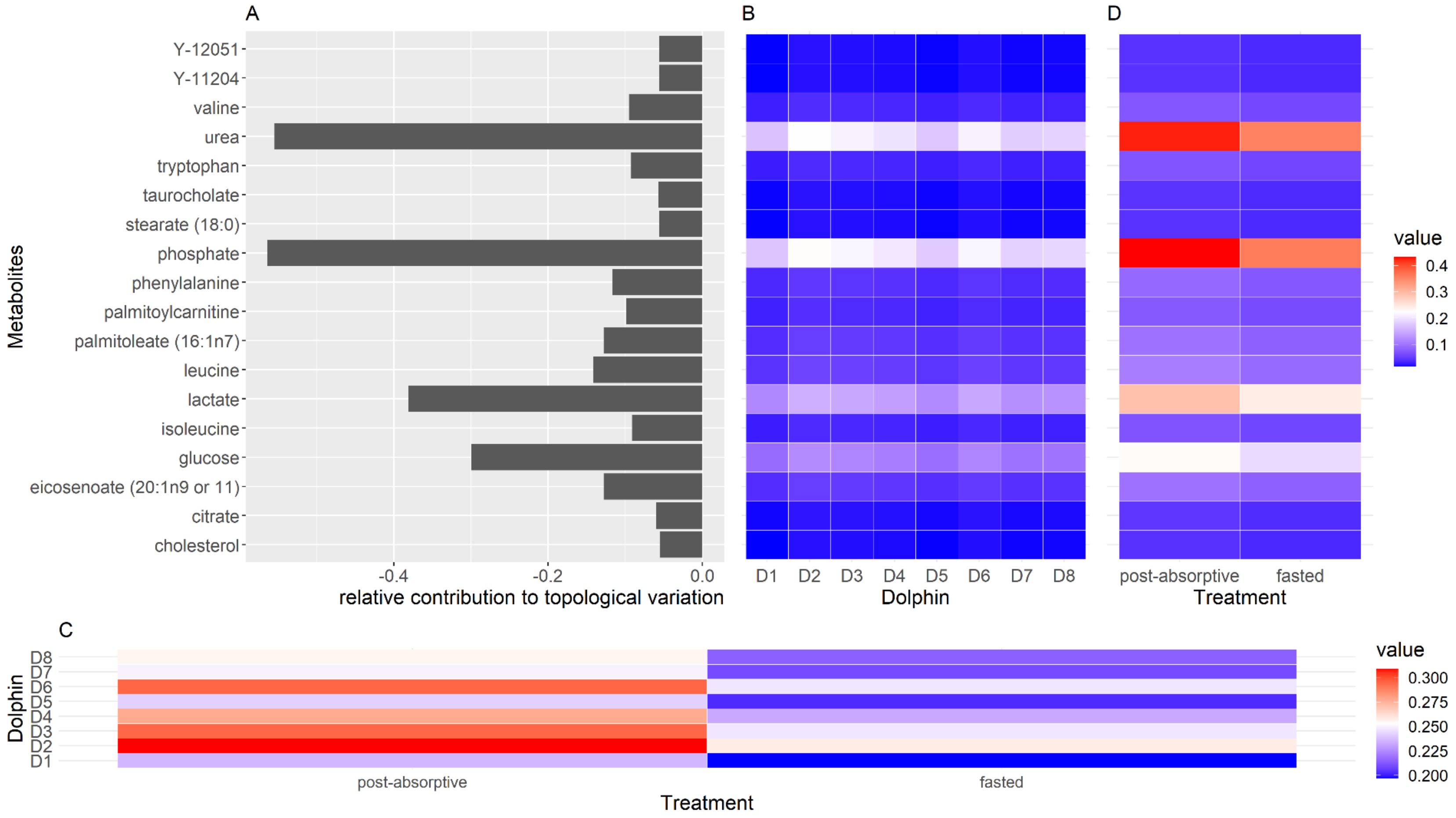

Journal of Experimental Biology • Supplementary information

Figure S2. Metabolite CMI networks (A: post-absorptive; B: fasted): nodes are metabolites, links represent the conditional mutual information between metabolite pairs (thickness represents the value), node size is the eigenvector centrality of metabolites and node colour corresponds to the main metabolic 'super-pathways' to which metabolites belong (see Figure 2 for legend).

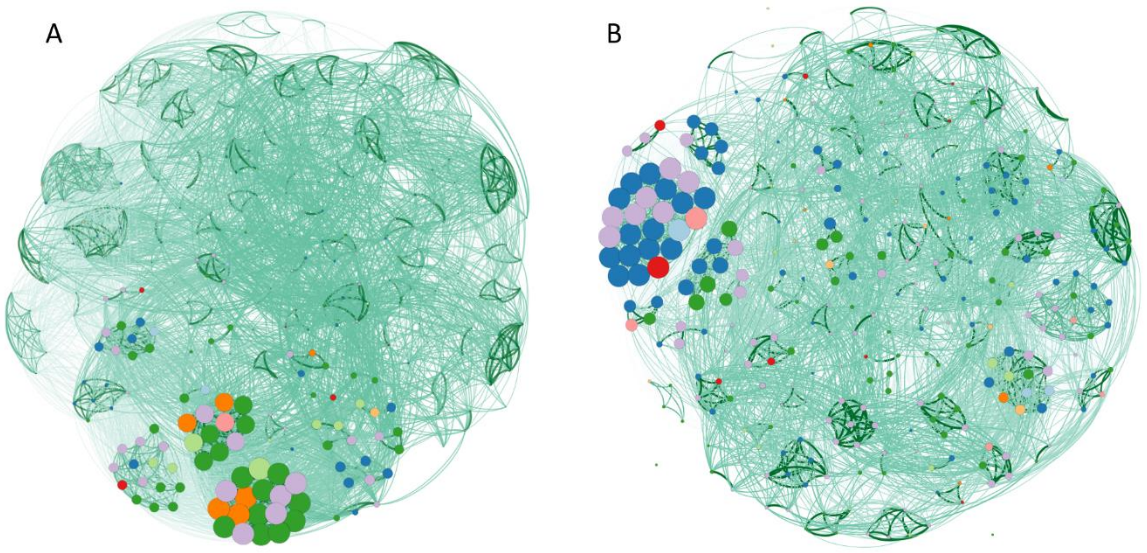

Figure S3. Change in module membership between post-absorptive (left, 12 modules) and fasted (right, 18 modules) conditions. The modules composed of nodes with largest eigenvector centrality are highlighted for post-absorptive (blue) and fasted (red) conditions, showing a dramatic change in membership. The modularity coefficient (Q_{module}) of each module is provided. Note, the proposed NDM has the largest Q_{module} (0.58).

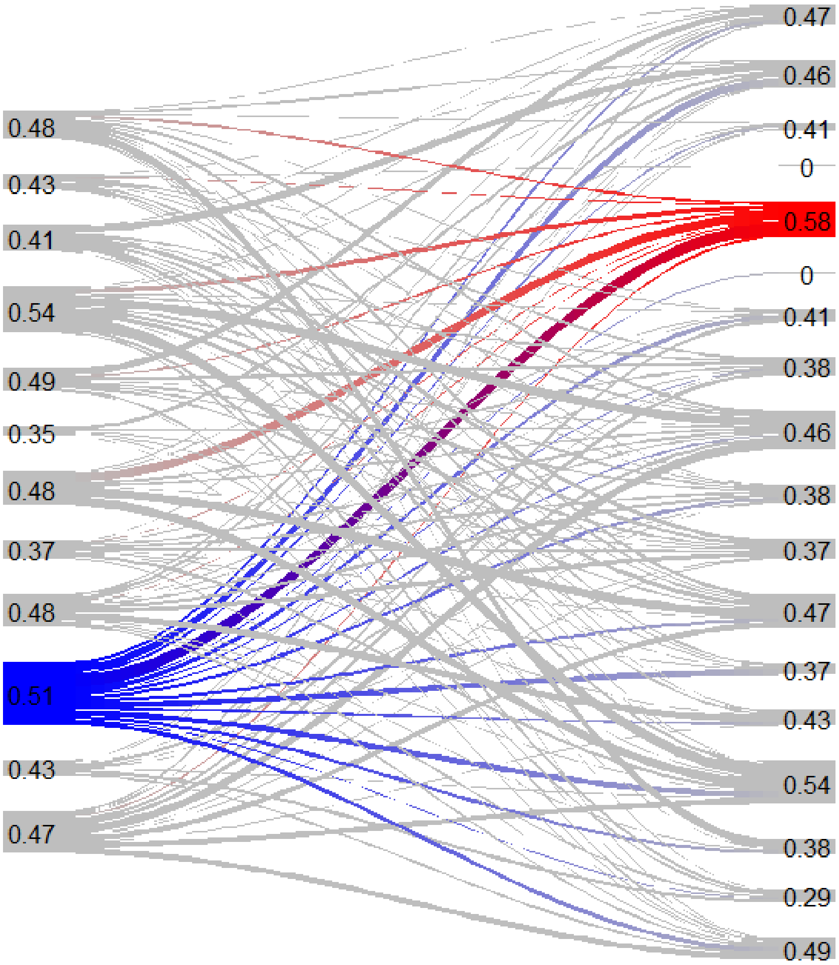

Journal of Experimental Biology • Supplementary information

Table S1. Intensity ratio – intensity fasted/intensity post-absorptive - for identified metabolites in the plasma of dolphins, along with biological super-pathway and sub-pathway membership. A value above 1 corresponds to an increase once fasted (highlighted in red) and a value below 1 corresponds to a decrease once fasted (highlighted in green). Significance of this ratio (significantly different from 1) is displayed with *p*-values and *q*-values, which are *p*-values adjusted for the false discovery rate, as performed by Metabolon.

Click here to download Table S1

Table S2. Biological functions putatively activated, including pathways to which they belong, their associated activation score (*Z*< -2: decrease, *Z*>2 increase), the number of relevant molecules associated with the functions identified in the data, and the *p*-value of the overlap. A *Z*-score of 2 is taken as a crude cut-off value to predict the activation of a function (predicted activation state column).

Click here to download Table S2

Table S3. List of the 32 metabolites belonging to the candidate Dynamic Network Marker, including Metabolon ID for those that could not be identified (beginning with Y-).

1-arachidonoylglycerophosphocholine*
1-docosahexaenoylglycerophosphocholine*
1-eicosadienoylglycerophosphocholine*
1-heptadecanoylglycerophosphocholine
1-linoleoylglycerophosphocholine
1-myristoylglycerophosphocholine
1-oleoylglycerophosphocholine
1-oleoylglycerophosphoethanolamine
1-palmitoleoylglycerophosphocholine*
1-palmitoylglycerophosphocholine
1-stearoylglycerophosphocholine
2-hydroxybutyrate (AHB)
2-oleoylglycerophosphocholine*
2-palmitoylglycerophosphocholine*
2-stearoylglycerophosphocholine*
3-methyl-2-oxobutyrate
fructose
heme*
oxaloacetate
pantothenate
stearoylcarnitine
Y-11497
Y-11550
Y-11561
Y-11809
Y-11812
Y-11949
Y-12051
Y-12421
Y-13426
Y-13619
Y-14562

Journal of Experimental Biology • Supplementary information
